# Editorial: New discoveries in bioengineering applied to vascular surgery

**DOI:** 10.3389/fsurg.2023.1293094

**Published:** 2023-10-13

**Authors:** P. Settembrini, S. Sultan, A. Settembrini

**Affiliations:** ^1^ Department of Biomedical and Clinical Sciences, L. Sacco University Hospital, Università Degli Studi di Milano, Milan, Italy; ^2^ Department of Vascular and Endovascular Surgery, Western Vascular Institute, University Hospital Galway, National University of Ireland, Galway, Ireland; ^3^ Galway Clinic, Doughiska, Royal College of Surgeons in Ireland and the National University of Ireland, Galway Affiliated Teaching Hospitals, Galway, Ireland; ^4^ CÚRAM-CORRIB-Vascular Group, National University of Ireland, Galway, Ireland; ^5^ Department of Cardio Thoracic and Vascular Diseases, Fondazione IRCCS Ca’ Granda Ospedale Maggiore Policlinico, Milan, Italy

**Keywords:** vascular surgery, artificial intelligence, graft, vascular biomaterial, saphenous graft

**Editorial on the Research Topic** New discoveries in bioengineering applied to vascular surgery

## 1. Introduction

The field of vascular medicine has witnessed the fifth cardiovascular revolution (1), thanks to the convergence and the synergistic relationship between vascular biomaterials, synthetic vascular neuronal networks, and artificial intelligence.

Healthcare has long been the beneficiary of technological advancements, with innovations consistently pushing the boundaries of what's possible in medical science. Among these remarkable developments, vascular bioengineering stands out as a beacon of hope, poised to revolutionize the treatment of cardiovascular diseases and usher in a new era of personalized medicine. This field has made substantial progress through the convergence of biology, engineering, and medical science, offering glimpses into a future where regenerative therapies, artificial organs, and minimally invasive procedures are commonplace.

These innovations have revolutionized the diagnosis and treatment of vascular diseases, paving the way for precision medicine tailored to individual patient needs (2).

### 1.1. The burden of cardiovascular diseases

Cardiovascular diseases (CVDs) represent a significant global health challenge, with conditions like Atherosclerosis, aneurysms, and ischemic disorders ranking among the leading causes of mortality. These diseases often necessitate invasive and high-risk interventions, such as open-heart surgeries or the implantation of synthetic materials. However, these conventional approaches are plagued by inherent issues, including the risk of rejection, limited durability, and the need for lifelong medication. Vascular bioengineering emerges as a promising solution to these long-standing problems. Vascular diseases, including atherosclerosis, aneurysms, and ischemic conditions, are among the leading causes of mortality worldwide. Traditional treatment approaches often involve open surgeries or the implantation of synthetic materials, which present various challenges, such as the risk of rejection, limited durability, and the need for lifelong medication. Vessel characteristics vary by size, and the bioengineering of human conduits must adjust to these requirements: the primary role and, therefore, the anatomic and biochemical design of large vessels is in the efficient transport of blood while vessels of smaller diameter gradually switch to performing more exchange functions in lower pressure, slower flow environment.

Repair, graft replacement, and reconstruction are the challenges for the expected benefits of bioengineering human tissues and blood vessels because the number of patients affected by peripheral arterial disease (PAD), requiring peripheral surgery, is steadily growing. PAD and vascular injuries, including civilian and military traumatic injuries, represent another area of the critical need for replacement vessels (3).

Vascular replacements started with autologous saphenous vein graft described in 1949, and in the following years prostethic grafts have been developed (4).

### 1.2. The promise of vascular bioengineering

#### 1.2.1. Regenerative therapies

One of the most exciting prospects in vascular bioengineering is the development of regenerative therapies. Stem cell research, for instance, has shown tremendous potential in growing functional blood vessels that can be tailored to individual patient needs. These regenerative approaches promise to replace damaged or diseased vessels with biologically compatible constructs, reducing the risks associated with synthetic materials.

#### 1.2.2. Artificial organs

Vascular bioengineering also holds the key to creating artificial organs that mimic the complex functions of the human circulatory system. These bioengineered organs, like artificial hearts and blood vessels, can potentially provide patients with life-saving solutions, reducing the waiting times and complications associated with organ transplantation.

#### 1.2.3. Minimally invasive procedures

Traditional surgical interventions often entail significant trauma and extended recovery periods. Vascular bioengineering, however, is driving the development of minimally invasive procedures. Techniques like catheter-based treatments and drug-eluting stents are becoming more sophisticated, allowing precise interventions with reduced patient discomfort and recovery times.

#### 1.2.4. Personalized medicine

One of the most profound impacts of vascular bioengineering is the move towards personalized medicine. By leveraging advancements in genomics and biotechnology, healthcare providers can tailor treatments to each patient's unique genetic makeup, optimizing outcomes and minimizing adverse effects.

## 2. Vascular biomaterials

Vascular biomaterials are engineered materials designed for compatibility with the human vascular system.

They have played a pivotal role in the development of advanced vascular therapies. These biomaterials can be divided into two categories: biological and synthetic.

Biological biomaterials, such as tissue-engineered grafts and decellularized matrices, offer the advantage of biocompatibility. They can support cell growth and tissue regeneration, making them valuable for repairing damaged vessels. On the other hand, synthetic biomaterials, like biodegradable polymers, provide mechanical strength and controlled degradation rates. They are often used for stent coatings, promoting healing while preventing restenosis.

## 3. Synthetic vascular neuronal networks

Synthetic vascular neuronal networks, a relatively new concept, involve creating artificial neural networks that mimic the autonomic nervous system's control over vascular functions. These networks integrate with the vascular system to monitor and regulate real-time blood flow, vasodilation, and vasoconstriction. They are particularly beneficial in managing conditions like hypertension and vascular insufficiencies.

These networks are designed to adapt to individual patient needs, ensuring precise control over vascular parameters. AI algorithms play a crucial role in optimizing the performance of these synthetic networks by continuously analyzing data from digital twins and sensors and then making necessary adjustments.

## 4. Artificial intelligence

Artificial Intelligence (AI) has emerged as a game-changer in the field of vascular medicine. Machine learning algorithms can process vast amounts of patient data, including medical records, imaging studies, and genetic information, to identify patterns and predict disease risks. This enables early diagnosis and personalized treatment plans.

AI-driven image analysis, such as in medical imaging and pathology, enhances the accuracy of vascular disease diagnosis. Moreover, predictive analytics help forecast disease progression, allowing for proactive interventions. AI-driven robotics and surgical assistance also contribute to the precision and safety of vascular procedures.

## 5. Precision vascular medicine

The synergy of vascular biomaterials, synthetic neuronal networks, and AI culminates in precision vascular medicine. Tailored treatment plans are created based on individual patient profiles, optimizing therapeutic outcomes while minimizing side effects.

Here are some key benefits:

1. Personalized Drug Delivery: Biomaterials can be used as drug carriers, releasing medications at specific locations within the vascular system, guided by synthetic networks and AI algorithms.
2. Targeted Therapies: AI analyzes patient data to identify optimal treatment strategies, considering genetic factors, lifestyle, and disease progression, resulting in targeted therapies.
3. Minimized Risks: Real-time monitoring and control by synthetic networks reduce procedural risks and enhance the safety of vascular interventions.
4. Improved Outcomes: Early diagnosis, precise treatment, and continuous monitoring lead to improved patient outcomes and quality of life.

Vascular grafts are usually made of synthetic polymers, animal and cadaveric tissues, or autologous and used with well-characterized outcomes, leaving areas of unmet need for the patients in terms of durability and long-term patency, susceptibility to infection, immunogenicity, inflammation, and mechanical failure. Vascular bioengineering aims to overcome these hurdles by leveraging cutting-edge techniques to create functional, biocompatible vascular replacements. One of the most promising areas within vascular bioengineering is tissue engineering.

One of the most significant challenges to the deployment of vascular grafts is their adoption by the host and their ability to remodel into new tissues. Several research strategies have been implemented to reduce infection rates, promote endothelialization, and inhibit inflammation. While some histological data demonstrate partial recellularization of xenografts, many clinical reports of explanted specimens provide evidence of inflammation, calcification, and neointima formation without cellularization of the implanted ECM (5).

Another facet of vascular bioengineering focuses on developing advanced prosthetics and implants.

Innovations such as bioresorbable stents that gradually dissolve after serving their purpose (6) innovative implants that monitor and respond to physiological changes, and tissue-engineered heart valves that mimic natural function are reshaping the landscape of cardiovascular interventions.

Minimally invasive procedures have become the cornerstone of modern medicine, offering reduced risks, shorter recovery times, and improved patient comfort. Vascular bioengineering contributes to this trend by creating catheters, delivery systems, and imaging techniques that enable precise interventions through small incisions. These advancements are extending the benefits of vascular treatments to a broader range of patients who may not have been candidates for invasive surgeries.

While the progress in vascular bioengineering is undeniably promising, challenges remain.

Ensuring the long-term durability and compatibility of engineered tissues and materials, addressing potential immune responses, and navigating regulatory pathways are all critical considerations. Graft failures due to loss of integrity, inflammation, and fibrosis have repeatedly been observed, often in environments experiencing mechanical stress or high pressures. Autologous grafts remain the preferred option for vessel replacement or repair, eliminating the risk of rejection presented by xenografts and allogenic vein grafts, resisting infection better than synthetic materials, and displaying mechanical properties most closely aligned with the conduit to be replaced. However, they face limitations concerning availability, risk of thrombosis due to damaged endothelium, intimal hyperplasia, and accelerated atherosclerosis. New research avenues focus on generating innovative materials that would decrease the risk of infection, thrombosis, and rejection and either undergo remodeling by the host or induce regeneration and repair of the host's tissues. Other two topics are relevant in the evolution of the vascular field: the use of robotics and endovascular devices and their relationship to the arterial wall.

The introduction of robotics represented another significant step forward for different surgical specialties, facilitating and improving the performance of minimally invasive surgery. Robot-assisted surgery has been brought into the area of vascular surgery to enhance laparoscopic vascular and endovascular skills, such as a relatively tricky manipulation of instruments and long suturing times for anastomoses and clamping of the aorta or pelvic arteries (7). It is well documented in the literature despite none of the systems described above having been employed on a widespread basis. The surgeon's movements are down-scaled into fine gestures, physiological tremor is eliminated, and the visualization is improved, thus simplifying those actions unachievable in traditional surgery. High costs and the lack of approval for use in the vascular field have contributed to their low popularity. However, concerns about addressing higher costs in favor of substantial health benefits for medical staff and patients should be considered.

The second aspect is critical of endovascular procedures. Endovascular procedures aim to create structural support to the arterial wall for a new path for the blood into the arteries, excluding aneurysms by the risk of enlargement and rupture or creating a new way in case of previous arterial occlusion.

Especially about the aneurysm treatment, the relationship between the endograft and the arterial wall can be understood in terms of the following key points: sealing and anchoring; integration and healing (it helps improve the long-term stability of the repair), minimization of blood flow (endograft alters the blood flow dynamics within the artery) and potential complications (treatment of certain vascular conditions, there can be complications related to the relationship between the graft and the arterial wall). Careful patient selection, proper sizing and positioning of the endograft, and regular follow-up are essential to minimize these risks.

The ideal design of the aortic endograft should resemble the native aorta in terms of its flexibility and hemodynamic impedance. The stent-graft polymers should be lightweight but strong, resilient, and capable of withstanding the impact of normal pulsatile high-flow arterial blood pressure. There is increasing evidence of adverse hemodynamic alteration post-TEVAR/EVAR. Interventionalists must respect the aorta as an active organ, not a mere conduit. The best solution in the short term could be to reduce the stented length of the aorta while, in the longer term, encouraging continuous improvement in stent-graft materials and design. The relationship between a vascular endograft and the arterial wall is a complex interplay of biomechanical factors, healing processes, and medical considerations. The success of endovascular procedures relies on the proper selection and placement of the endograft, as well as careful management and monitoring to ensure optimal patient outcomes.

The intelligent endoprosthesis will adapt to prevent tissue ingrowth into its’ microstructure, preventing rigidity and maintaining distensibility. Therefore, the Smart endoprosthesis will retain the ability to expand in systole and collapse in diastole. After implantation, it returns the elastic recoil to the heart, creating an almost standard aortic flow curve (8).

Vascular bioengineering represents a transformative force in cardiovascular medicine, offering solutions once confined to science fiction. As researchers push the boundaries of knowledge and technology, we can anticipate a future where vascular diseases are treated with unprecedented precision, patients experience an improved quality of life, and healthcare becomes increasingly personalized.

The potential to replace damaged vessels with bioengineered constructs, create artificial organs, and perform minimally invasive procedures heralds a brighter and healthier future for individuals affected by cardiovascular diseases. Through the intersection of biology, engineering, and medical science, vascular bioengineering is paving the way for a healthcare landscape that is more effective, patient-centered, and hopeful.

## 6. Conclusion

The convergence of vascular biomaterials, synthetic vascular neuronal networks, and artificial intelligence have ushered in a new era of precision vascular medicine.

Vascular bioengineering is poised to reshape the landscape of cardiovascular medicine, offering solutions that were once confined to science fiction. We anticipate a future where vascular diseases are treated with precision, patients experience improved quality of life, and healthcare becomes increasingly personalized.

This innovative approach offers personalized solutions for patients with vascular diseases, enhancing diagnostic accuracy and treatment efficacy. As these technologies continue to advance, the future of vascular healthcare promises even more remarkable breakthroughs, ultimately improving the lives of countless individuals suffering from vascular conditions.
